# Scarlet Fever Epidemic in China Caused by *Streptococcus pyogenes* Serotype M12: Epidemiologic and Molecular Analysis

**DOI:** 10.1016/j.ebiom.2018.01.010

**Published:** 2018-01-11

**Authors:** Yuanhai You, Mark R. Davies, Melinda Protani, Liam McIntyre, Mark J. Walker, Jianzhong Zhang

**Affiliations:** a State Key Laboratory for Infectious Disease Prevention and Control, National Institute for Communicable Disease Control and Prevention, Chinese Center for Disease Control and Prevention, Beijing, China; b Department of Microbiology and Immunology, Peter Doherty Institute for Infection and Immunity, The University of Melbourne, Melbourne, Victoria, Australia; c School of Public Health, The University of Queensland, Brisbane, QLD, Australia; d School of Chemistry and Molecular Biosciences, Australian Infectious Diseases Research Centre, The University of Queensland, Brisbane, QLD, Australia

**Keywords:** Scarlet fever, Group A *Streptococcus*, Mainland China, Epidemiological data, Genomic evolution

## Abstract

From 2011, Hong Kong and mainland China have witnessed a sharp increase in reported cases, with subsequent reports of epidemic scarlet fever in North Asia and the United Kingdom. Here we examine epidemiological data and investigate the genomic context of the predominantly serotype M12 *Streptococcus pyogenes* scarlet fever isolates from mainland China. Incident case data was obtained from the Chinese Nationwide Notifiable Infectious Diseases Reporting Information System. The relative risk of scarlet fever in recent outbreak years 2011–2016 was calculated using the median age-standardised incidence rate, compared to years 2003–2010 prior this outbreak. Whole genome sequencing was performed on 32 *emm12* scarlet fever isolates and 13 *emm12* non-scarlet fever isolates collected from different geographic regions of China, and compared with 203 published *emm12 S. pyogenes* genomes predominantly from scarlet fever outbreaks in Hong Kong (n = 134) and the United Kingdom (n = 63). We found during the outbreak period (2011–2016), the median age-standardised incidence in China was 4.14/100,000 (95% confidence interval (CI) 4.11-4.18), 2.62-fold higher (95% CI 2.57-2.66) than that of 1.58/100,000 (95% CI 1.56-1.61) during the baseline period prior to the outbreak (2003 − 2010). Highest incidence was reported for children 5 years of age (80.5/100,000). Streptococcal toxin encoding prophage φHKU.vir and φHKU.ssa in addition to the macrolide and tetracycline resistant ICE-*emm*12 and ICE-HKU397 elements were found amongst mainland China multi-clonal *emm12* isolates suggesting a role in selection and expansion of scarlet fever lineages in China. Global dissemination of toxin encoded prophage has played a role in the expansion of scarlet fever *emm12* clones. These findings emphasize the role of comprehensive surveillance approaches for monitoring of epidemic human disease.

## Highlights

• The study used all epidemiological data from 1950 to 2016, and describe increased incidence levels for the current outbreak.
• Using global *emm12* scarlet fever isolate genome sequences, the multiclonal nature of the outbreak was confirmed.
• Global surveillance of GAS toxin and drug resistance mobile genes in the scarlet fever outbreak is necessary.

Our study provides a detailed report of scarlet fever epidemiology and genomic analysis for mainland China since the 2011 outbreak began. We also provide a comprehensive comparison of the genomic relationship of scarlet fever outbreak *emm12* isolates from China, Hong Kong and the United Kingdom, countries experiencing an unparalleled re-emergence of scarlet fever. Our observations implicate an important role for GAS toxin and drug resistance related mobile genes in the outbreak and reveal different evolutionary patterns, and identify common themes relating to the acquisition of toxin carrying prophage elements. This work emphasizes the importance of comprehensive nationwide surveillance to track scarlet fever, GAS *emm* types, exotoxin-encoding prophage and antibiotic resistance genes in a global context.

## 1 Introduction

Scarlet fever ranked as one of the most severe infectious diseases prior to the widespread use of antibiotics in the 1940s. Scarlet fever is caused by the Gram positive bacterium *Streptococcus pyogenes* (group A *Streptococcus*, GAS) which is also responsible for several other diseases including suppurative pharyngitis and tonsillitis, impetigo and erysipelas, cellulitis, toxic shock and necrotizing fasciitis. Repeated infection also triggers the autoimmune sequelae rheumatic fever, rheumatic heart disease and acute post-streptococcal glomerulonephritis (Walker et al., 2014). Scarlet fever outbreaks began in 2011 in mainland China, Hong Kong, Vietnam and South Korea. *S. pyogenes emm12* is most frequently isolated from scarlet fever cases in China (Luk et al., 2012, Park et al., 2017, Tse et al., 2012, Yang et al., 2013, You et al., 2013, Wong and Yuen, 2012, Wu et al., 2013). In 2014, a scarlet fever epidemic has been reported in the United Kingdom caused by *S. pyogenes* isolates of *emm3*, *emm4*, *emm1* and *emm12* (Basetti et al., 2017, Chalker et al., 2017, Lamagni et al., 2017, Turner et al., 2016).

Several factors have been proposed to play a role in triggering these outbreaks, including changing bacterial population structure, enhanced capacity of GAS to cause scarlet fever through gene acquisition, changes in host herd immunity, meteorological factors and potential association between the bacteria and co-infection with an as yet unidentified factor that may predispose the host to scarlet fever caused by GAS. The underlying cause(s) of these outbreaks remain unresolved (Ben Zakour et al., 2015, Chalker et al., 2017, Coleman, 2016, Davies et al., 2015, Duan et al., 2017, Lee et al., 2017, Soderholm et al., 2017, Tse et al., 2012). Previous studies have shown that the predominantly *emm12* GAS isolates responsible for the Hong Kong outbreak have acquired mobile genetic elements including integrative and conjugative elements encoding tetracycline and macrolide antibiotic resistance and prophage encoding superantigens SSA, SpeC and the DNase Spd1 (Tse et al., 2012, Davies et al., 2015). Related mobile genetic elements were also identified in *emm1* GAS causing scarlet fever in Hong Kong (Ben Zakour et al., 2015).

Despite the epidemic lasting over 6 years, there has not been a comprehensive nationwide epidemiological description at the epicenter of the North Asia outbreak. In this study, we provide an up to date epidemiological characterization of the scarlet fever outbreak in China. We have also collected both historical and outbreak-related *emm12* GAS isolates from distinct geographic regions of mainland China, and employed whole genome sequencing to gain insight into genomic evolution, virulence profile and antimicrobial resistance gene carriage of scarlet fever causing GAS.

## 2 Materials and Methods

### 2.1 Epidemiological Data Collection and Analysis

The epidemiological scarlet fever case data used in this study was obtained from the Chinese Nationwide Notifiable Infectious Diseases Reporting Information System (established in 2003). Population data was obtained from the National Bureau of Statistics of China (http://data.stats.gov.cn – accessed 20.12.17). The age-specific incidence of scarlet fever for mainland China was extracted from this surveillance system by selecting years from 2003 to 2016. Direct age-standardisation was performed using the age-specific incidence rates (0–14, 15–64, 65 +) of scarlet fever for each year, standardised to the most recent (2015) population data. 95% confidence intervals were calculated using the exact method. Age-adjusted risk ratios (RR) and 95% confidence intervals (CI) were calculated by dividing the age-standardised median incidence rates to examine relative differences of incidence of scarlet fever in 2011–2016, compared to 2003–2010. For the analysis of scarlet fever spatial distribution, we focus on data reported after 2011. The geographic incidence data were extracted from 2011 to 2016. The age and occupation data for scarlet fever incidence were extracted and shown for the year 2016, after a comparison of this data was made with the data from 2011 to 2015; 2016 data was found representative for the six outbreak years. We collected all available data for scarlet fever incidence in China. After the new People's Republic of China was founded in 1949, this data was reported from 1950. We collected the incidence data from 1950 to 2002 from the Health Statistics Yearbook of the National Health and Family Planning Commission (http://www.nhfpc.gov.cn/zwgkzt/tjnj/list.shtml). The above spatial, temporal and population distribution data were analyzed using Excel and Stata 14.0. This manuscript was written in accordance with RECORD guidelines.

### 2.2 GAS Isolates From Mainland China

A national molecular epidemiologic investigation was performed from 2004 to 2011, to characterize *emm* types, virulence factors and antimicrobial resistance of strains circulating in both northern areas of China with high scarlet fever incidence and southern areas with low incidence. Throat swab samples were collected from patients clinically diagnosed as scarlet fever or pharyngitis. Scarlet fever cases were defined as patients who presented with fever (> 38 °C), sore throat, ‘sandpaper-like’ rash on the trunk and limbs/extremities, and ‘strawberry-like’ tongue. In total, forty-five GAS *emm12* isolates from mainland China were investigated (Supplementary Table 1). These include 32 isolated by throat swab from diagnosed scarlet fever cases collected from areas with high incidence of scarlet fever in 2011 when the outbreak of scarlet fever first began. These areas included representative strains from Heilongjiang, Tianjin, Beijing and Shenyang. Eight historic isolates were also included for genomic analysis. We also included 139 publicly available *emm12* genomes reported from Hong Kong which is close to Guangdong province and here used to represent the south east of China, 1 from Lebanon (Ibrahim et al., 2016), 63 from the United Kingdom (Chalker et al., 2017), 2 from the USA and 3 from Australia (Davies et al., 2015). Collectively, a total of 248 *emm12* genomes were characterized in this study.

### 2.3 Genome Sequencing of *emm12* Isolates

*S. pyogenes* isolates were cultivated on Columbia agar base supplemented with 5% sheep blood and incubated at 37 °C for 24 h. DNA was extracted using a Qiagen Mini kit. GAS *emm12* isolates were sequenced using Illumina Hiseq2000. Paired-end libraries with 500-bp insertions were generated, and the read lengths were 90 bp. For each isolate, 450 Mb of high-quality raw data was generated. Publicly available genome data were obtained from GenBank or the European Nucleotide Short Read Archive and shredded to an estimated 75 × coverage of paired-end 100 bp reads using SAMtools wgsim for inclusion in phylogenetic comparisons.

### 2.4 Phylogenetic Analysis

Sequencing reads were mapped to the 1,908,100 bp Hong Kong *emm12* scarlet fever reference genome HKU16 (Tse et al., 2012) using the genome aligner SMALT v0.7.4 (http://www.sanger.ac.uk/resources/software/smalt/). The minimum base call quality to call a single nucleotide polymorphism (SNP) was set at 50, and the minimum mapping quality to call a SNP was set at 30 (Harris et al., 2010). Reads aligning to regions of the HKU16 genome pertaining to repeat sequences and prophage regions were excluded from phylogenetic analyses as previously defined (Davies et al., 2015). To remove further phylogenetic ambiguities, regions of high SNP density within the *emm12* population were identified with Gubbins (Croucher et al., 2015). Using the combination of these approaches, a total 321,164 bases (16.8%) of the HKU16 reference genome was excluded, resulting in an alignment of 1,586,936 bp. A total of 2637 SNPs was used to determine the phylogenetic structure of the global *emm12* population. Maximum-likelihood analysis of consensus alignments was conducted using the AVX version of RAxML v8.2.8 (Stamatakis, 2006). The general time-reversible model with gamma correction performed with 100 bootstrap random re-samplings to assess support for the maximum-likelihood phylogeny.

### 2.5 Assessment of Virulence, Antibiotic Resistance, and Mobile Genetic Elements Carriage

Carriage of antibiotic resistance genes and exotoxin superantigen genes was assessed using ABRicate (https://github.com/tseemann/abricate) at a cut-off of 75% of sequence identity from draft SPAdes v3.11.1 genome assemblies. Assessment of mobile genetic elements (MGE) repertoire was determined through a read mapping approach against a database of 17 *emm12* MGE (10 prophage sequences and 9 ICE elements) as identified from population analyses of Hong Kong scarlet fever *emm12* (Davies et al., 2015). Reads were mapped with BWA v0.7.16 and depth counted with Samtools depth v1.6 for bases with a Phred score > 20. MGEs were considered present if 90% of the sequence had 10 × coverage.

### 2.6 Ethical Considerations

The National Institute for Communicable Disease Control and Prevention, China Center for Disease Control approved this study.

### 2.7 Data Access

Illumina sequence reads of 45 mainland China *emm12* GAS sequenced in this study were submitted to the Sequence Read Archive (SRA) of GenBank database under the Bioproject accession number PRJNA416675. Individual accession numbers for each genome are provided in Supplementary Table 1.

## 3 Results

### 3.1 Incidence of Scarlet Fever Across the Past Sixty Years in China

**Fig. 1:**
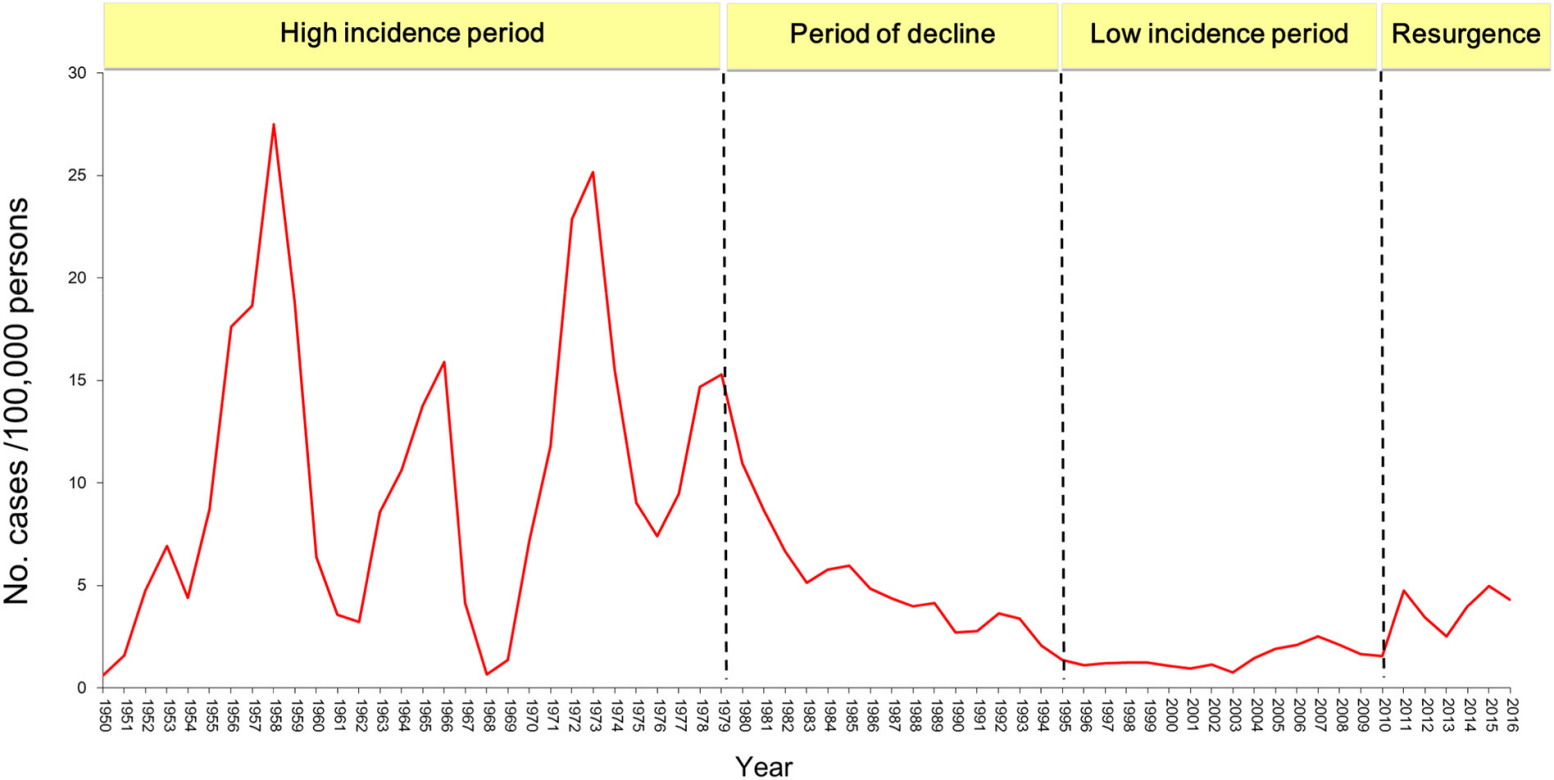
Scarlet fever incidence in China over the period 1950–2016.

From reported data on scarlet fever incidence over the past 66 years, four different stages can be observed. Between 1950 and 1979, there was a high incidence of disease (average 10.54/100,000) with a peak value approximately every 6 years. The highest peak appeared in 1958 (27.51/100,000). The low incidence in 1950 is quite likely due to under reporting, as the new people's republic of China was only founded in October 1949, and thus disease reporting was only being established at that time. Scarlet fever incidence between 1980 and 1994 then decreased (average value 4.58/100,000). A period of low disease incidence spanned 1995–2010 (average value 1.46/100,000). From 2011, China has undergone an unexpectedly sharp increase of scarlet fever cases (2011–2016 median 4.16/100,000), with peak incidence years in 2011 (63,878 cases) and 2015 (68,249 cases). The last time that the incidence reached the same level as the highest point in 2011–2016 (2015 4.96/100,000) was the year 1986 (4.84/100,000). (Fig. 1, Supplementary Table 2).

During the outbreak period (2011–2016), the median age-standardised incidence in China was 4.14/100,000 (95% Confidence interval (CI) 4.11–4.18), 2.62-fold higher (95% CI 2.57–2.66) than the median age-standardised incidence rate of 1.58/100,000 (95% CI 1.56–1.61) during the baseline period prior to the outbreak (2003–2010). As the data for incidence by age are not available for the year 2003 and 2016, age-standardised incidence rates between 2004 and 2015 are shown in Table 1.

**Table 1:** Annual incidence of scarlet fever (2004–2016).

Year	Number of cases	Population total (per 10,000)	Crude IR (per 100,000)	Age-standardised IR^a^ (95% CI) (per 100,000)
2004	19,024	129,988	1.46	1.14 (1.12–1.16)
2005	25,068	130,756	1.92	1.58 (1.56–1.61)
2006	27,620	131,448	2.1	1.78 (1.76–1.80)
2007	33,488	132,129	2.53	2.18 (2.15–2.20)
2008	27,782	132,802	2.09	1.84 (1.82–1.87)
2009	22,068	134,091	1.65	1.49 (1.47–1.51)
2010	20,876	134,091	1.56	1.55 (1.53–1.57)
2011	63,878	134,735	4.74	4.76 (4.72–4.80)
2012	46,459	135,404	3.43	3.44 (3.41–3.47)
2013	34,207	136,072	2.51	2.53 (2.50–2.56)
2014	54,247	136,782	3.97	3.97 (3.94–4.01)
2015	68,249	137,462	4.96	4.96 (4.93–5.00)
2016	59,282	138,271	4.29	N/A

Notes: IR = incidence rate.

a Age standardised incidence rates were calculated using the 2015 China population as the standard.

### 3.2 Seasonal Epidemic Pattern

**Fig. 2:**
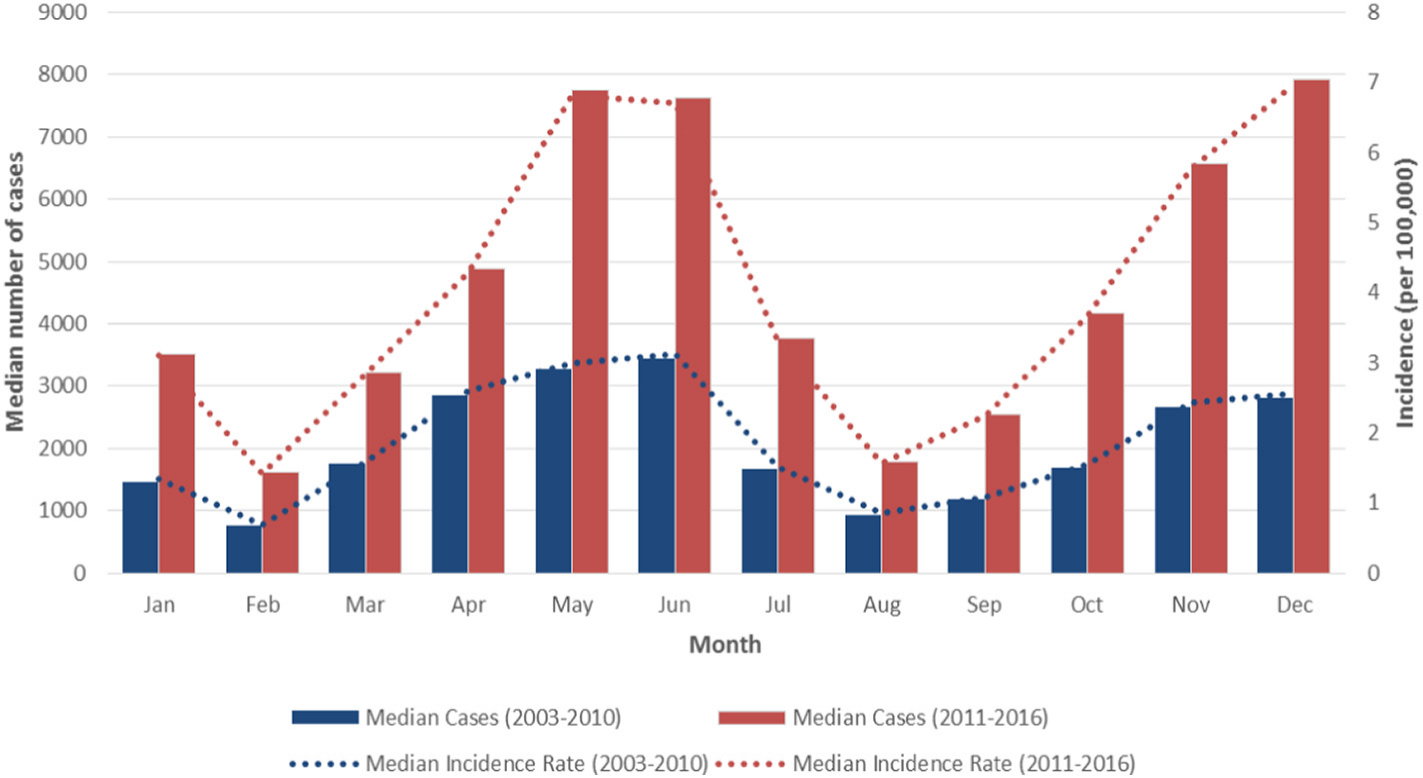
Monthly reported scarlet fever cases and incidence across the last fourteen years in China (2003–2010 and 2011–2016).

There are two peaks periods of incidence. The first peak occurs during May and June (early summer), with the second peak occurring in December (winter). The seasonal epidemic pattern was similar between 2003 and 2010 and 2011–2016, but dramatically amplified in the 2011–2016 period. (Fig. 2, Supplementary Table 3).

### 3.3 Geographic Distribution of Scarlet Fever Cases in Mainland China (2011–2016)

**Fig. 3:**
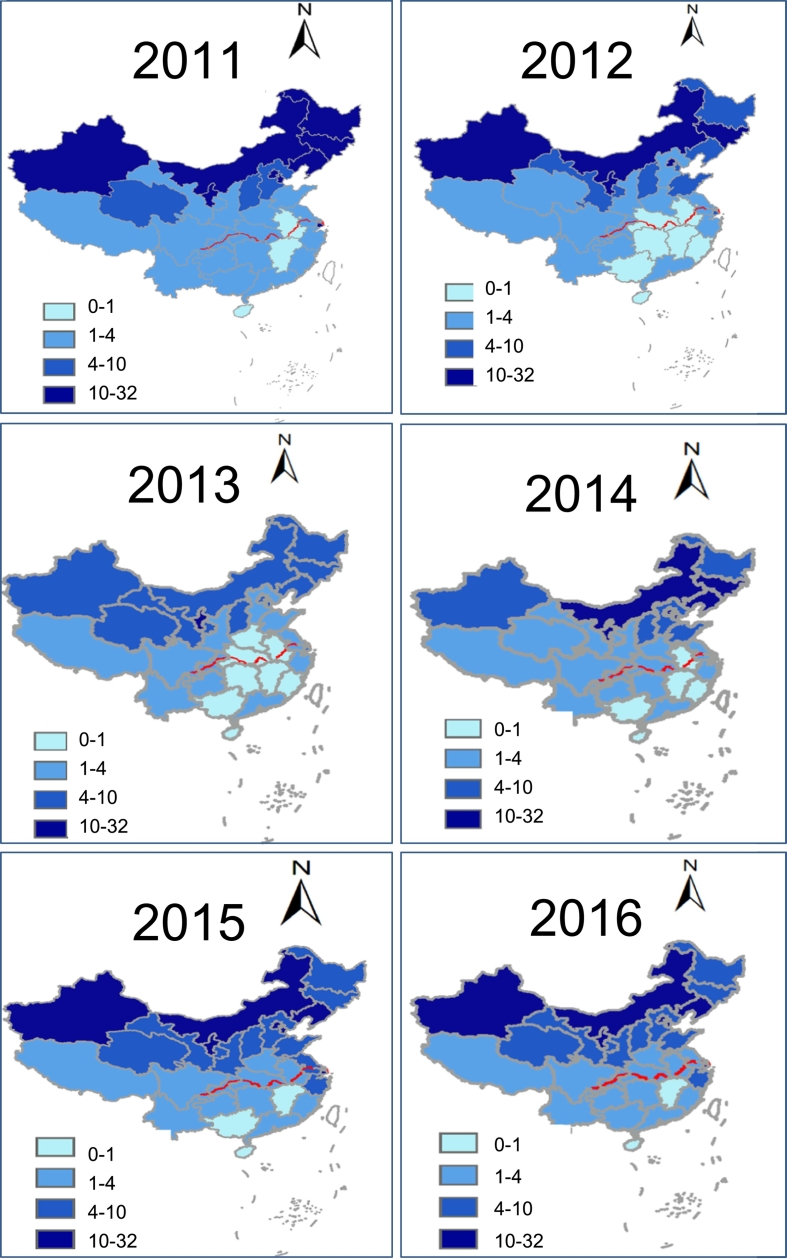
Reported scarlet fever incidence according to geographic region in China (2011–2016).

To take a geographic view of scarlet fever incidence during the current outbreak, we examined the incidence data of all 31 provinces of mainland China from 2011 to 2016 (Supplementary Table 4). The data for each year was geographically matched to the map of China according to the incidence value for each province. Accordingly, all provinces were divided into four categories with different incidences. Areas with incidence between 10 and 32 per 100,000 people were defined as high incidence regions, such as Beijing, Shanghai and northern provinces (Fig. 3; dark blue). Provinces with an incidence below 1 per 100,000 were defined as low incidence regions, and included Hainan, Jiangxi and Guangxi (Fig. 3; light blue). Areas with incidences between 1 and 4 and 4–10 were designated intermediate incidence regions, and included Yunnan, Jiangxi and Guangdong. Almost all high incidence regions are located north of the Yangtze River which flows from west to east across China (Fig. 3).

### 3.4 Demographic Characteristics Within the Population Relating to Occupation and Age

**Fig. 4:**
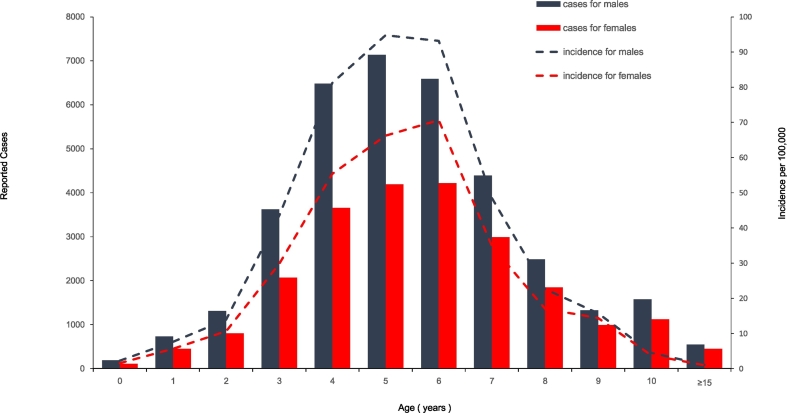
Age, gender and incidence of reported scarlet fever cases in China for 2016.

Review of the population data for scarlet fever from 2011 to 2016, shows each year displaying a similar incidence profile. Here we elaborate on the population data for 2016. Case numbers of males was 1.55 fold higher than females (Fig. 4). The age group from 3 to 9 years old accounted for 83% of total cases. Five-year old children had the highest incidence with 82.86 per 100,000. Children attending kindergarten account for 44% of total cases, while school students accounted for 35%. Age groups including the kindergarten and school students accounted for 79% of total cases. Children under 3 years old that are not enrolled in kindergarten account for 18% of total scarlet fever cases (Table 2).

**Table 2:** 2016 scarlet fever reported cases based on occupation/category.

	Category/occupation	No. of cases
Children	Kindergarten children	26,283 (44.34%)
	Students	21,195 (35.75%)
	Non-kindergarten children	11,251 (18.98%)
Adults	Rural subsistence farmers	188 (0.32%)
	Staying at home/unemployed	99 (0.17%)
	Workers	65 (0.11%)
	Clerks	63 (0.11%)
	Other	47 (0.08%)
	Business services	36 (0.06%)
	Teachers	21 (0.04%)
	Migrant workers	10 (0.02%)
	Medical profession	8 (0.01%)
	Catering employees	6 (0.01%)
	Retirees	6 (0.01%)
	Waiters	3 (0.005%)
	Herders	1 (0.002%)

### 3.5 Population Genetics of Epidemic Scarlet Fever *emm12* GAS Isolates

**Fig. 5:**
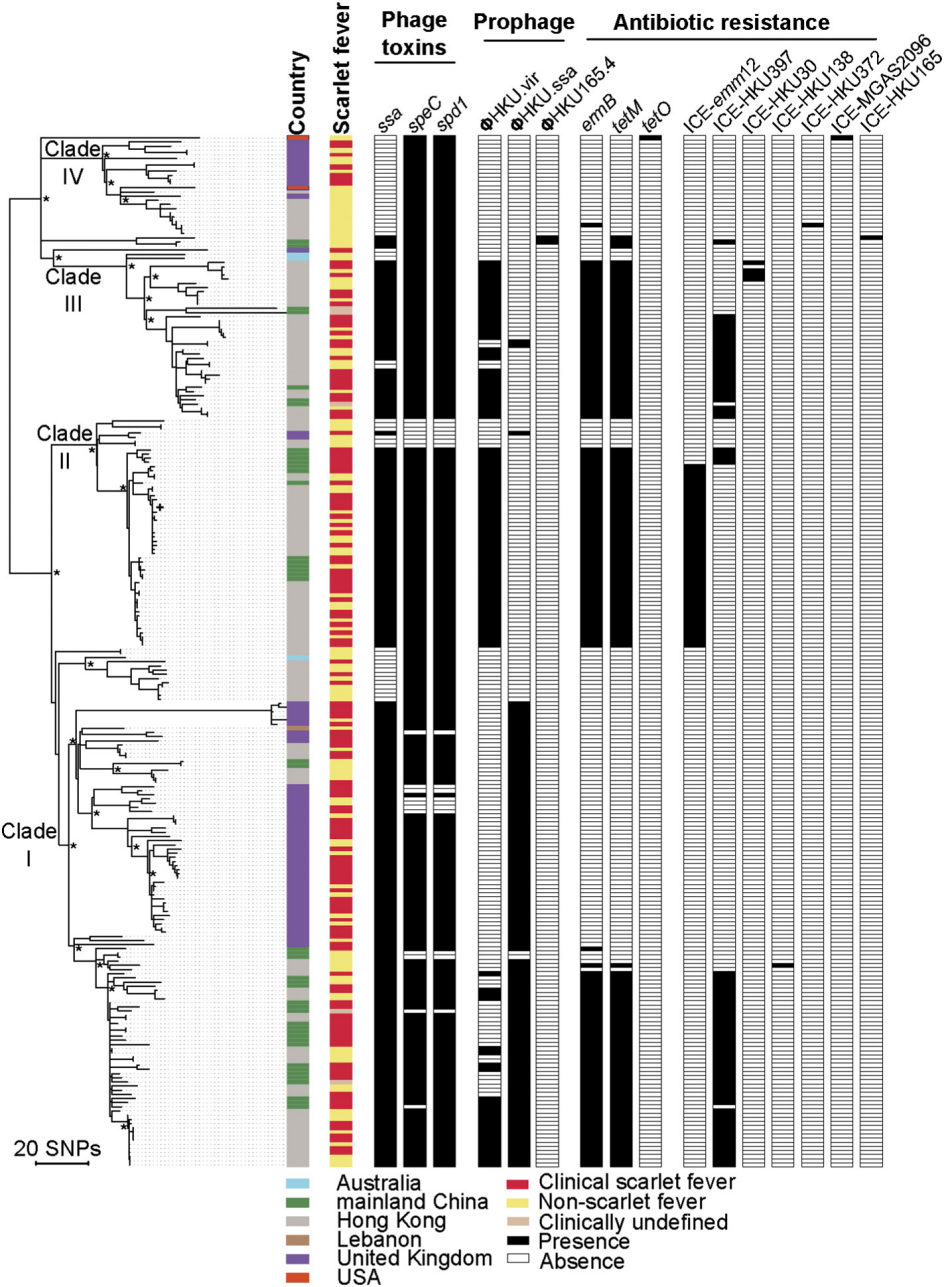
Midpoint-rooted maximum-likelihood phylogenetic tree of 248 GAS *emm12* isolates based on 2637 polymorphisms after mapping to the Hong Kong scarlet fever reference genome HKU163 and removal of confounding genomic regions (see methods). Phylogenetic location of the HKU16 genome is represented by the cross. Previously defined phylogenetic clades of the Hong Kong *emm12* lineage, termed Clades I-IV, are shown on major branches (Davies et al., 2015). Asterisks represent major nodes with > 95% bootstrap support. Terminal nodes are coloured by country of origin and clinical scarlet fever association. Relative distribution of selected virulence genes, prophage elements, antimicrobial resistance genes and integrative conjugative elements (ICE) that have previously been linked to the emergence of scarlet fever clones in Hong Kong(Davies et al., 2015) are displayed.

To assess the genomic relationship of scarlet fever associated *emm12* isolates within different geographical regions we undertook a population genomics study of 248 genome sequences, 45 of which were sequenced from mainland China (32 clinical scarlet fever isolates) as part of this study. Isolates were primarily from 2011 and 2012, with several isolates included from 2004 to 2007 to provide temporal context. Also included were published datasets from Hong Kong (Davies et al., 2015) (134 isolates, 42% clinical scarlet fever) and the United Kingdom (Chalker et al., 2017) (63 isolates, 71% clinical scarlet fever) which are two areas also reporting a significant upsurge of epidemic scarlet fever. To provide wider geographical context, we also included publicly available *emm12* genome sequences from USA (n = 2), Australia (n = 3) and Lebanon (n = 1). A total of 2637 core genome polymorphisms were identified after mapping of the genome sequences to the 2011 Hong Kong scarlet fever isolate HKU16 (Davies et al., 2015). The genomic structure was assessed by maximum likelihood phylogenetic analyses which showed that the overall structure of the phylogenetic tree was similar to our previous findings from Hong Kong, with the presence of at least four major genetic lineages (Fig. 5). Mainland China isolates clustered into three clades (clade I-III) and overall shared a close genetic relationship with those from Hong Kong. 87% of mainland China isolates (36/47) cluster into clade I and clade II, together with isolates from Hong Kong. Clade IV does not contain mainland China isolates, and this clade does not contain Hong Kong scarlet fever isolates as described in our previous study^13^. Scarlet fever isolates were distributed amongst isolates from other clinical states supporting our observations in Hong Kong that the outbreak is not phylogenetically restricted. In contrast, phylogenetic comparison of the Hong Kong and mainland China *emm12* isolates to the UK *emm12* strains revealed that the United Kingdom isolates primarily reside within distinct sub-lineages of clade I (40/63, 70%) and clade IV (12/63, 19%). This suggests that while the UK *emm12* strains share a common ancestral relationship to the China clades, they have since evolved geographically independently. Therefore, the evolution of scarlet fever clones in the United Kingdom and China cannot be explained by the intercontinental movement of a single scarlet fever clone.

### 3.6 Screening for Exotoxin and Drug Resistance Genes and Mobile Genetic Elements

Streptococcal superantigens have been linked to the classic immunopathology and disease etiology of scarlet fever patients. Indeed, we have recently inferred that the acquisition of exotoxin genes such as *ssa* and *speC* carried on prophages, and antibiotic resistance genes found on integrative conjugative elements, have been linked to the expansion of *emm12* scarlet fever associated clades in Hong Kong (Davies et al., 2015, Ben Zakour et al., 2015). All 45 mainland China isolates contain the *ssa* gene and 91% carried the *speC* gene. The previously identified prophage ΦHKU.vir from Hong Kong *emm12* outbreak reference strain HKU16 carrying the superantigen genes *ssa* and *speC* and the DNase gene *spd1*, was identified in 51% of mainland China isolates, predominantly belonging to clades II and III, while ΦHKU.ssa from the reference strain HKU360 carrying the superantigen gene *ssa* was also carried by 51% of mainland China isolates. Five isolates from mainland China carried both ΦHKU.vir and ΦHKU.ssa, commensurate with clade I related isolates from Hong Kong. Examination of the superantigen profiles within the 63 United Kingdom *emm12* isolates revealed high carriage of *spe*C and *spd*1 (82% and 84% respectively). Carriage of *ssa* was high, 49/63 (77%), and was phylogenetically restricted to the Clade I-like lineage. Only ΦHKU.ssa was identified in the United Kingdom *emm12* population, with no isolates carrying a ΦHKU.vir phage, suggesting that dissemination of ΦHKU.ssa may have played an important role in the evolution of scarlet fever associated lineages in both countries. Of the 8 *emm12* genomes representing isolates from Lebanon, Australia and USA, only the Clade I isolate SP1LAU from Lebanon contained *ssa* which was carried on ΦHKU.ssa.

Macrolide and tetracycline resistance in GAS has emerged as a major health concern in China (You et al., 2013). In the 45 genome sequences from mainland China, we observed carriage of *ermB* and *tetM* to be 87% and 96% respectively. Analysis of the genomic context of these resistance genes revealed that ICE-*emm*12 (20% positive) and ICE-HKU397 (62% positive) were the primary vehicles of antibiotic resistance in GAS *emm12* within mainland China. In contrast, no United Kingdom *emm12* isolates carried any of the multidrug resistant genes or their associated ICE elements that were common to *emm12* GAS from China.

## 4 Discussion

Since 2011, resurgence of scarlet fever has been reported in mainland China, Hong Kong, South Korea, Taiwan, Vietnam and the United Kingdom. Scarlet fever outbreaks had caused significant disease burden in China up until the early 1980s, but then receded as a cause of disease burden and morbidity. However, from 2011 a sharp increase in reported cases began and the incidence of scarlet fever in China remains high as of the end of 2016 (326,322 total cases). Our study utilizes the Chinese CDC Nationwide Notifiable Infectious Diseases Reporting Information System to investigate scarlet fever epidemiological and incidence data for the Chinese population.

From the 1950s to 1980s, the incidence of scarlet fever was high compared to the subsequent decades of disease decline. Although the 2011 outbreak has witnessed a 2.7 fold increase (4.75/1.76) compared to the average level of the previous three decades, disease incidence is not as high as that recorded prior to the 1980s. Nonetheless, close attention needs to be paid to the current epidemic. The China CDC continues to investigate the underlying reasons for the outbreak against the setting of an otherwise rapid development of the Chinese economy and improvement in the national health system, and with increasing frequency of domestic and international population mobility in recent years. Many factors may possibly interact in this outbreak, including environmental factors, climate, living standards, population movement, host population genetics and herd immunity; some of these may potentially have influenced changes in geographic distribution of cases during 2011–2016.Epidemic areas and low incidence areas are temporally changing in this period, especially in the south-east regions of mainland China, where several provinces are observed to shift between low and intermediate incidence areas. An acknowledged limitation of this study is that we were unable to perform a time-series analysis to examine the underlying drivers of scarlet fever. Pathogen surveillance for GAS *emm* type is not universally undertaken across mainland China, making it difficult to comprehensively investigate *S. pyogenes* serotype shifts before and during the outbreak. Nonetheless, in some large cities including Beijing and Shanghai, which both encompass high incidence areas, a serotype shift from *emm12* to *emm1* has been observed in some districts, though *emm12* is still the dominant type in most areas (Cui et al., 2014).

Based on the age of the reported scarlet fever cases, children of 3 to 9 years old account for 83% of total cases. This is quite similar to a recent report of the epidemiology of scarlet fever in England, which shows 87% of cases are individuals under the age of 10 years (Lamagni et al., 2017). This correlates with the observation that this age group is at the stage of establishing protective immune function and may lack herd immunity to prevent GAS infection.

We have also generated genome sequence data of 32 *S. pyogenes emm12* scarlet fever isolates from different geographic regions of mainland China, and compared these data with 203 published *S. pyogenes emm12* scarlet fever genomes from Hong Kong and the United Kingdom. This analysis allows us to examine and report, for the first time, the phylogenetic context of *emm12* scarlet fever isolates from different global locations. Previous genomic studies have found that Hong Kong *emm12* outbreak isolates have two important mobile genetic elements: prophage (ΦHKU.vir and ΦHKU.ssa) encoding streptococcal exotoxins (SSA, SpeC and Spd1), and integrative and conjugative elements including ICE-*emm*12 encoding multidrug resistance (tetracycline and macrolides). Though these horizontally transferred genomic elements are hypothesized to be associated with the outbreak in Hong Kong, there has not been a detailed analysis for their distribution and genomic context in mainland China GAS isolates or the scarlet fever associated *emm12* lineage from the United Kingdom. Mainland China contains 1.35 billion people, representing approximately 20% of the world population, and the country contains many diverse geographic environments. We therefore chose to explore the genomic characteristics of *emm12* GAS outbreak isolates from mainland China. Here we have integrated 45 *emm12* mainland China isolates in comparison to 134 *emm12* Hong Kong isolates and 63 *emm12* isolates from the United Kingdom (Chalker et al., 2017) to investigate the evolutionary pattern of the prevailing *emm12* lineage in geographically distinct regions where scarlet fever outbreaks are ongoing. We found that the majority of mainland China *emm12* scarlet fever isolates fall into two previously described clades (clade I and clade II) while the United Kingdom *emm12* isolates form genetically distinct sub-populations within clades I and IV. Unlike the United Kingdom isolates, none of the mainland China isolates fell into clade IV, which has been proposed previously to not be associated with scarlet fever. Hong Kong is located near the south-east corner of mainland China, close to Shenzhen city and Guangzhou city in regions of low or medium disease incidence. It would be valuable to expand molecular epidemiologic surveillance to these areas of mainland China to compare strain characteristics. Examination of the superantigen profile and associated prophage carriage revealed that ΦHKU.vir is strongly associated with clade I-III isolates from mainland China and Hong Kong while ΦHKU.ssa is associated with the clade I sub-lineage from the United Kingdom which was associated with 75% of *emm12* related scarlet fever cases. The United Kingdom clade I lineage also clustered with an *emm12* isolate from a pharyngitis patient in Lebanon (Ibrahim et al., 2016) suggesting that *ssa* associated *emm12* clones and their mobile elements may be more geographically dispersed than previously recognized. We, and others, have previously shown than *ssa* antigen carriage is over-represented in scarlet fever isolates from other GAS *emm* types isolated during the outbreak such as the M1T1 lineage (Chalker et al., 2017, Ben Zakour et al., 2015). Monitoring of the dissemination of *ssa* containing GAS clones and prophage elements such as ΦHKU.ssa in a global context is warranted.

In summary, this is a detailed report of scarlet fever epidemiology and genomic analysis for mainland China since the 2011 outbreak. This is also the first analysis that compares the genomic relationship of scarlet fever outbreak *emm*12 isolates from China and the United Kingdom, two countries experiencing an unparalleled re-emergence of scarlet fever. Our observations implicate an important role for GAS toxin and drug resistance related mobile genes within outbreak *emm12* scarlet fever clones, and find a different evolutionary pattern between the United Kingdom and China, yet linked by a common theme relating to the carriage of related toxin carrying mobile genetic elements. This work emphasizes the importance of comprehensive nationwide surveillance to track scarlet fever, GAS *emm* type drift, exotoxin-encoding prophage genes as well and antibiotic resistance genes for *S. pyogenes* isolates in a global context.

## Funding

This work was supported by a grant from the State Key Laboratory of Infectious Disease Prevention and Control (SKLID) (2014SKLID102) of the Chinese Center for Disease Control and Prevention, and the National Health and Medical Research Council of Australia (APP1126805). The funders played no role in study design, data collection, data analysis, interpretation, or writing of the report.

The following are the supplementary data related to this article.

Supplementary material 1 Image 1

Supplementary material 2 Image 2

Supplementary material 3 Image 3

Supplementary material 4 Image 4

Supplementary Fig. 1Identification of putative recombinogenic regions based on the whole genome alignment of 248 GAS *emm12* genomes. Maximum-likelihood phylogeny as represented in Fig. 5 is represented on the left. The HKU16 reference genome is represented at the top of the figure in orange. Regions of the HKU16 genome identified as mobile genetic elements, repeat regions or high SNP density are indicated by the grey boxes at the top of the figure with genomic locations of HKU16 related prophage and integrative conjugative elements indicated. Polymorphisms located within these ‘grey’ regions were excluded for phylogenetic analysis on the basis of confounding vertically evolved polymorphisms. Red and Blue regions indicate potential location of recombined ‘blocks’ for each taxa in the tree with blue referring to segments unique to a single taxa. Larger recombination blocks are largely limited to mobile genetic elements. Blocks were identified using Gubbins (Croucher et al., 2015) and visualized using Phandango (Hadfield et al., 2017). Image 5
