# Supplementary figures and images for: Scarlet Fever Epidemic in China Caused by Streptococcus pyogenes Serotype M12: Epidemiologic and Molecular Analysis
Source: eBioMedicine. 2018 Jan 11;28:128–35. doi: 10.1016/j.ebiom.2018.01.010 (PMC5835554; doi:10.1016/j.ebiom.2018.01.010)

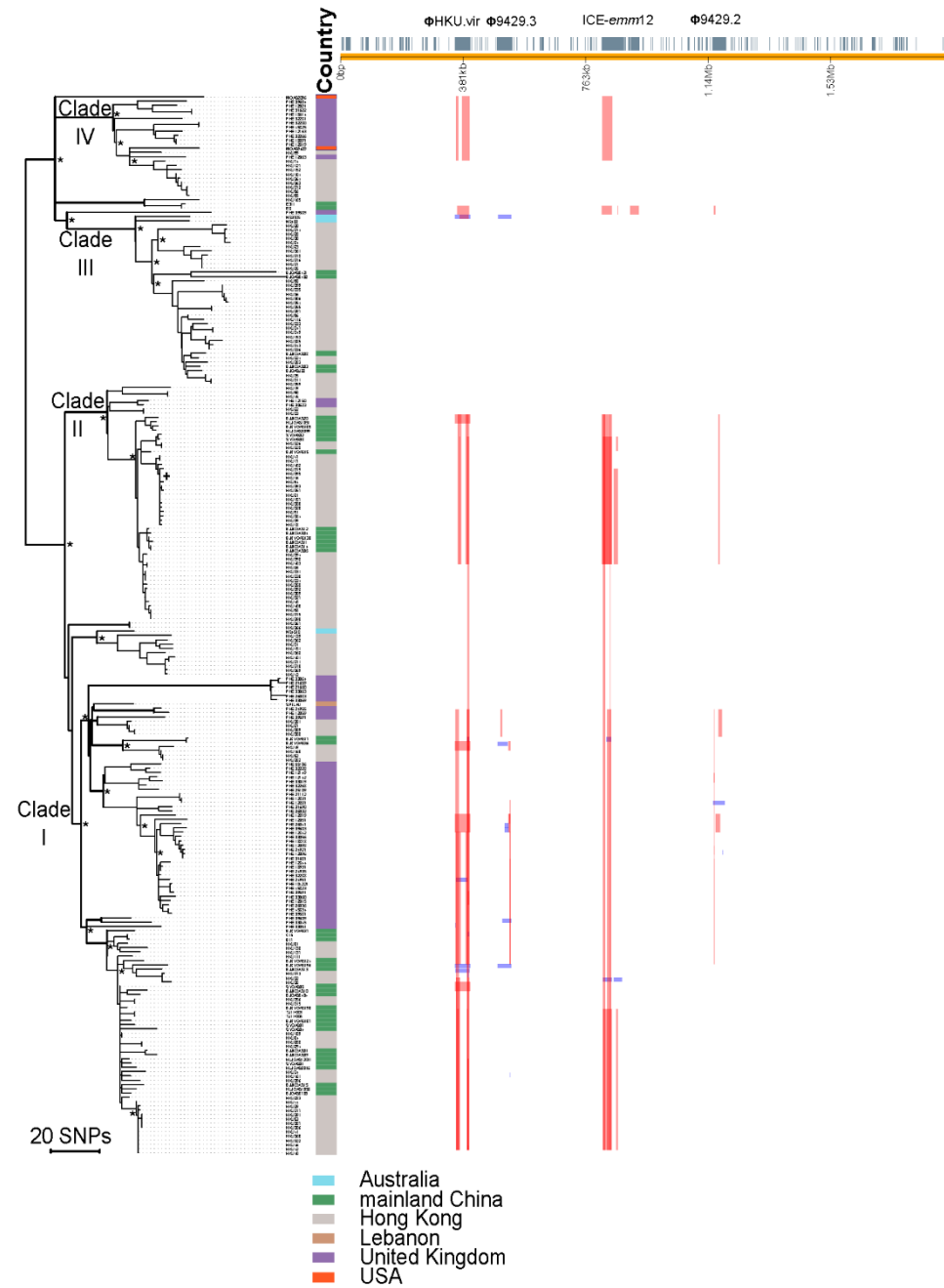

Supplement: Supplementary file 5 — Supplementary Fig. 1Identification of putative recombinogenic regions based on the whole genome alignment of 248 GAS emm12 genomes. Maximum-likelihood phylogeny as represented in Fig. 5 is represented on the left. The HKU16 reference genome is represented at the top of the figure in orange. Regions of the HKU16 genome identified as mobile genetic elements, repeat regions or high SNP density are indicated by the grey boxes at the top of the figure with genomic locations of HKU16 related prophage and integrative conjugative elements indicated. Polymorphisms located within these ‘grey’ regions were excluded for phylogenetic analysis on the basis of confounding vertically evolved polymorphisms. Red and Blue regions indicate potential location of recombined ‘blocks’ for each taxa in the tree with blue referring to segments unique to a single taxa. Larger recombination blocks are largely limited to mobile genetic elements. Blocks were identified using Gubbins (Croucher et al., 2015) and visualized using Phandango (Hadfield et al., 2017). [file mmc5.pdf]
